# Tabotamp^®^, Respectively, Surgicel^®^, Increases the Cell Death of Neuronal and Glial Cells In Vitro

**DOI:** 10.3390/ma13112453

**Published:** 2020-05-28

**Authors:** Sandra Leisz, Marie-Luise Trutschel, Karsten Mäder, Christian Scheller, Christian Strauss, Sebastian Simmermacher

**Affiliations:** 1 Department of Neurosurgery, Medical Faculty, Martin Luther University Halle-Wittenberg, 06120 Halle (Saale), Germany; Sandra.leisz@uk-halle.de (S.L.); Christian.scheller@uk-halle.de (C.S.); christian.strauss@uk-halle.de (C.S.); 2 Institute of Pharmacy, Faculty of Biosciences, Martin Luther University Halle-Wittenberg, 06120 Halle (Saale), Germany; marie-luise.trutschel@pharmazie.uni-halle.de (M.-L.T.); Karsten.Maeder@pharmazie.uni-halle.de (K.M.)

**Keywords:** oxidized regenerated cellulose, Tabotamp^®^, Surgicel^®^, pH, cell death

## Abstract

Oxidized regenerated cellulose (ORC) is an approved absorbable hemostat in neurosurgery, and contains 18–21% carboxylic acid groups. This modification leads to a low pH in aqueous solutions. Therefore, the aim of study was to analyze the pH-dependent effects of the ORC Tabotamp^®^ on astrocytes, Schwann cells, and neuronal cells in vitro to investigate whether Tabotamp^®^ is a suitable hemostat in cerebral eloquent areas. The ORC-dependent pH value changes were measured with (i) a pH meter, (ii) electron paramagnetic resonance spectroscopy, using pH-sensitive spin probes, and (iii) with fluorescence microscopy. Cell lines from neurons, astrocytes, and Schwann cells, as well as primary astrocytes were incubated with increasing areas of Tabotamp^®^. Cytotoxicity was detected using a fluorescence labeled DNA-binding dye. In addition, the wounding extent was analyzed via crystal violet staining of cell layers. The strongest pH reduction (to 2.2) was shown in phosphate buffered saline, whereas culture medium and cerebrospinal fluid demonstrated a higher buffer capacity during Tabotamp^®^ incubation. In addition, we could detect a distance-dependent pH gradient by fluorescence microscopy. Incubation of Tabotamp^®^ on cell monolayers led to detachment of covered cells and showed increased cytotoxicity in all tested cell lines and primary cells depending on the covered area. These in vitro results indicate that Tabotamp^®^ may not be a suitable hemostat in cerebral eloquent areas.

## 1. Introduction

Oxidized cellulose has been described as an absorbable hemostatic material in 1943 [1]. Cellulose is a homopolysaccharide [2] that can either remain non-regenerated with frayed unorganized fibers or be regenerated to form smooth organized fibers [3]. The treatment with dinitrogen tetroxide converts the hydroxyl groups into carboxyl acid groups [4]. This oxidization makes cellulose susceptible to glycosidases, and gives oxidized cellulose its hemostatic and bactericidal properties [3].

Oxidized regenerated cellulose (ORC), for example Surgicel^®^ (brand name USA) or, respectively, its European equivalent Tabotamp^®^ (brand name EU), is often used in neurosurgery to handle small vessel bleeding and oozing [5]. Surgicel^®^ generates an artificial brownish clot by producing acid hematin [6,7]. Additionally, mechanical actions like tamponade, swelling, and gel formation are described [8]. Aggregation through platelet activation was not observed for oxidized cellulose [9]. Wagner and colleagues published a hemostatic effectiveness ranking, where Surgicel^®^ showed the lowest hemostatic effect [10].

In contrast to the reported complete dissolution of ORC within six weeks [1], Surgicel^®^ was still detectable subcutaneously and in the brain 30 and 36 days after implantation [11,12]. It is recommended to remove Tabotamp^®^ at the end of surgery to avoid pressure effects on the surrounding tissue by swelling of the material (manufacturer’s instructions) [8]. In case of intracerebral tumor surgery, removal of Tabotamp^®^ increases the risk of rebleeding; therefore, the absorbable ORC is often left in place. Other studies found local effects, which are described as follows: A relevant drop of pH in normal saline, phosphate buffer solution and pooled human plasma after incubation with ORC had been shown [3,13,14]. Experiments on rat sciatic nerve showed that application of oxidized cellulose lowers subperineural pH to around 3 and kept it below 4 for two hours [15]. Additionally, incubation with oxidized cellulose solved in medium (2 mg/mL and 10 mg/mL) reduces the neurite outgrowth of cultured rat dorsal root ganglion neurons [14]. Furthermore, the incubation with ORC reduces the proliferation and migration of fibroblasts [13]. After the implantation of ORC into the parietal lobe of cats, Frantz described a mild tissue reaction in five out of eight cases and a comparable tissue reaction after implantation of temporal muscle, which was taken as a control. But two of the ORC samples were additionally soaked in thrombin. This means an intense tissue reaction or necrosis was documented in 50% of the remaining cases [1]. With respect to the small number of existing studies in contrast to the wide spread use, we systematically analyzed the effects of Tabotamp^®^ on the pH in phosphate buffered saline (PBS), cerebrospinal fluid (CSF), and cell culture medium (DMEM) by three independent methods (pH electrode, electron paramagnetic resonance spectroscopy, and fluorescence microscopy). With pH-sensitive fluorescence microscopy, we aimed to measure the acidity not only in bulk, but also on the ORC (Tabotamp^®^) surface to demonstrate the existence of a local pH gradient. There is currently no study examining acidity on different cells of the nervous system. Therefore, we studied the cytotoxicity of astrocytes, Schwann cells and neurons depending on increasing amounts of Tabotamp^®^. Schwann cells are the myelin sheath forming glia cells of the peripheral nervous system. Because of their function in enveloping and electric isolation of nerve cell axons, they play an important role in neuroprotection, nerve regeneration, and modulation of neuromuscular synaptic activity. Astrocytes are glia cells of the central nerve system and involved in repair mechanisms and building the physical structure of the brain. Due to their regenerative functions, we analyzed, beside neuronal cells, the Tabotamp^®^-dependent cell death of Schwann cells and astrocytes. In addition, the local effects of Tabotamp^®^ in comparison to non-oxidized cellulose on the different cell monolayers were analyzed.

## 2. Results

### 2.1. Tabotamp^®^ Decreases pH Value in Phosphate Buffered Saline, Cell Culture Medium and Cerebrospinal Fluid

**Figure 1:**
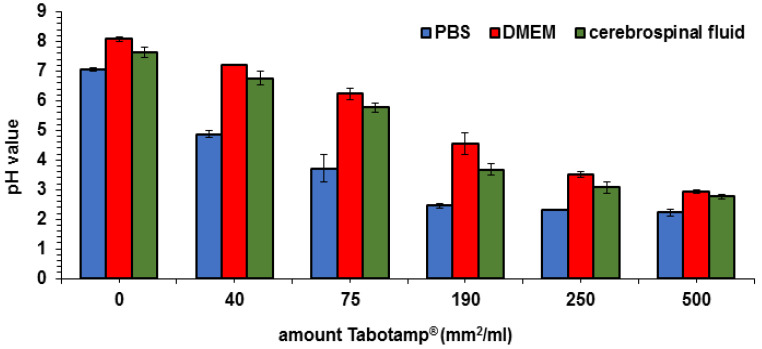
Impact of Tabotamp^®^ load and media composition on pH values determined with pH electrode. The diagram shows the means and standard deviations of three independent experiments.

The pH value was measured via pH electrode with increasing areas of Tabotamp^®^ per milliliter phosphate buffered saline (PBS), medium, or cerebrospinal fluid. The incubation of Tabotamp^®^ with PBS showed the strongest reduction of pH (Figure 1). Already the amount of 75 mm^2^ (5.1 mg) Tabotamp^®^ per mL led to a reduction of pH from 7.1 ± 0.05 to 3.7 ± 0.48. A pH value of 2.2 ± 0.12 was reached with 500 mm^2^ Tabotamp^®^ per mL PBS. The culture medium (Dulbecco’s Modified Eagle’s Medium, DMEM) contains 44 mM sodium bicarbonate and 10% fetal calf serum (FCS). Because of the higher protein concentration of DMEM and cerebrospinal fluid (CSF), the buffer capacity was increased compared to PBS. In DMEM, the pH was reduced from 8.1 ± 0.07 to 6.2 ± 0.21 and in cerebrospinal fluid from 7.6 ± 0.18 to 5.8 ± 0.17 during incubation with 75 mm^2^ Tabotamp^®^ per mL. The amount of 500 mm^2^ Tabotamp^®^ per mL caused strong acidic pH values of 2.9 ± 0.07 for DMEM and 2.8 ± 0.09 for CSF (Figure 1).

### 2.2. Analysis of pH via Electron Paramagnetic Resonance (EPR) Spectroscopy

**Figure 2:**
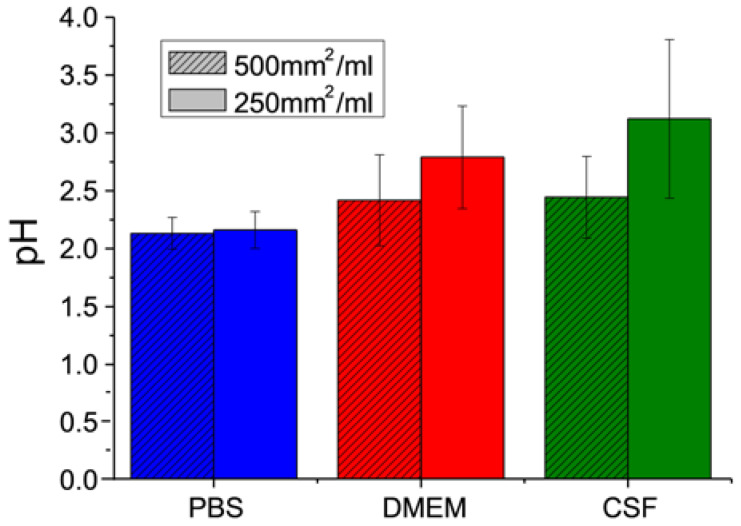
Analysis of oxidized regenerated cellulose (ORC)-dependent pH in phosphate buffered saline (PBS), supplemented culture medium (DMEM), and cerebrospinal fluid (CSF) determined with electron paramagnetic resonance (EPR) spectroscopy. Results of the pH measurements with the EPR probe HM (1 mmol/L) in 100 µL or 200 µL solution and 50 mm^2^ (3.4 mg) Tabotamp^®^. The diagram shows the means and standard deviations of three independent experiments.

The measurable pH range of 2,2,3,4,5,5-hexamethyl-imidazoline-1-oxyl (HM) is between pH 2 and 7 (see calibration curve Figure S1). At pH 2 and below, the nitroxyl radical is fully protonated and the hyperfine splitting a_N_ reaches its lower limit. Tabotamp^®^ in PBS shows this a_N_ at a concentration of 500 mm^2^/mL (Figure 2). DMEM and CSF buffer the acidity of the Tabotamp^®^ more than PBS, thereby the pH decreases to 2.4 ± 0.4 for both. The mixture of 250 mm^2^/mL Tabotamp^®^ with PBS, DMEM, or CSF results in a pH of 2.2 ± 0.2, 2.8 ± 0.5, and 3.1 ± 0.7, respectively.

### 2.3. Alterations in pH Value Focus on Tabotamp^®^ Itself and Surrounding Area 

**Figure 3:**
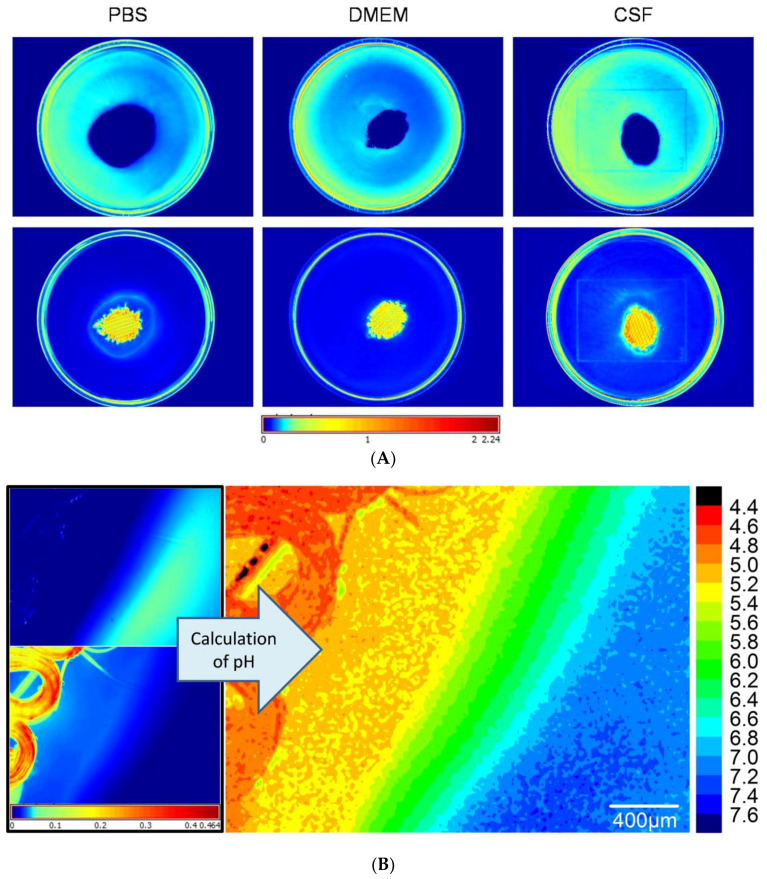
Analysis of ORC-dependent local pH in PBS, DMEM, and CSF determined with pH-sensitive fluorescence imaging and microscopy. (**A**) 5 cm diameter petri dish with SNARF in the medium and 95 mm² Tabotamp^®^ in the middle. The intensity distribution of the neutral spectrum is shown at the top and the acidic spectrum at the bottom. The colors were set with the Histogram eq. mode and the color map “jet”. The scale is in parts of the maximum intensity. The blue stands for low intensity and red for high intensities. The knitting structure is shown in orange. (**B**) Fluorescence microscope picture of Tabotamp^®^ in CSF. The left side shows the neutral and the acidic spectrum distribution. The right side presents the calculated pH as described in methods. The pH was calculated with a sigmoidal calibration curve (Figure S2).

Ninety-five mm^2^ Tabotamp^®^ was placed in PBS, DMEM, or CSF with or without 4.8 µg/mL SNARF-4F 5-(and-6)-carboxylic acid (SNARF) filled petri dish and the fluorescence signals were measured. The spectra of SNARF in acidic and neutral environment differ in the emission wavelength of the peak maximum. This offers the possibility to discriminate the local acidity depending on the intensity distribution. The fluorescence images show the intensity distribution of the neutral spectrum of the SNARF (Figure 3A, top) and the intensity distribution of the acidic spectrum of SNARF (Figure 3A, bottom). The highest intensity of the acidic SNARF spectrum was measured in the direct contact with Tabotamp^®^ and the surrounding area. Within a distance of 1 mm, neutral pH values were reached. A closer look gives the fluorescence microscope image in Figure 3B. In contrast to Figure 3A, the right scale illustrates pH values. The areas of the Tabotamp^®^ fibers have a pH below 5 (smaller pH values cannot be detected because SNARF is completely protonated at pH 5). The pH values are low in the fiber proximity and increase to neutral values (blue area) over a distance of 1 mm to the pH value of the medium. Therefore, the existence of pH gradients was detected.

### 2.4. Tabotamp^®^ Incubation Leads to Detachment of Cell Monolayer

**Figure 4:**
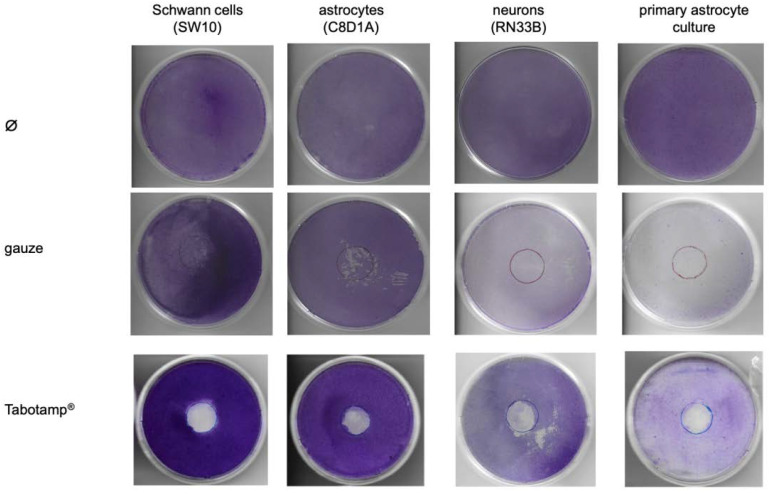
Impact of materials and cell type on cell adherence/detachment. The cells were cultivated in 10 cm diameter dishes to complete confluence and then incubated with Tabotamp^®^ or gauze. Non-oxidized cellulose (gauze) served as a control to investigate the direct influence of ORC-induced pH-reduction. After 24 h, the cells were fixed with methanol and stained with crystal violet as described in method section. The figure shows one representative dish out of at least three independent experiments.

Cells were grown to complete confluent monolayer in 10 cm diameter culture dishes and incubated with 380 mm^2^ Tabotamp^®^ or sterile gauze for 24 h. Then, culture dishes were stained with crystal violet solution as described in the method section. The staining showed a complete removal of cells after contact with Tabotamp^®^ (Figure 4) as opposed to cells incubated with gauze. The cells incubated with gauze showed only slight mechanical detachment of cells. The effect of Tabotamp^®^ was independent of the analyzed cell type. Tabotamp^®^ incubation damaged the cell layer of Schwann cells, neuronal cells and immortalized or primary astrocytes in a comparable extent, but no alterations on the surrounding confluent cell layer was detected. 

### 2.5. Tabotamp^®^ Incubation Leads to Cell Death of Schwann Cells, Neuronal Cells and Astrocytes

**Figure 5:**
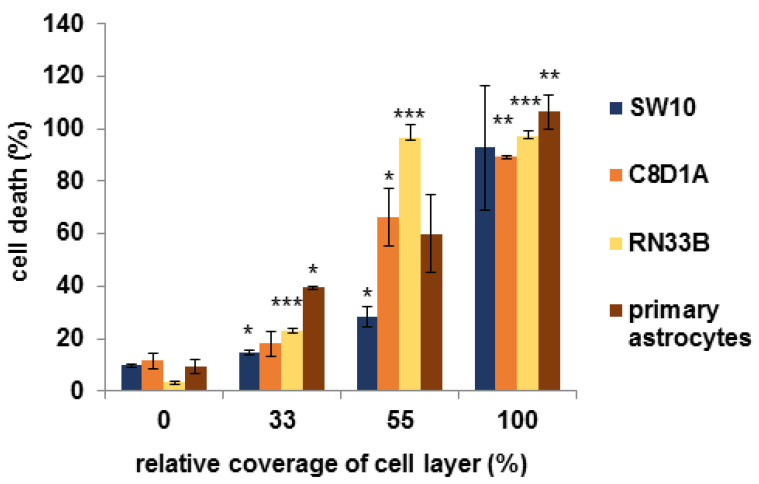
Tabotamp^®^-dependent cell death of Schwann cells, neuronal cells and astrocytes. 5 × 10^3^ Schwann cells (SW10), respectively, 1 × 10^4^ astrocytes (C8D1A; primary astrocytes) or neuronal cells (RN33B) were seeded in triplicates in black flat 96 well plates and incubated for 24 h. Then, 0, 11, 19, and 34 mm^2^ of Tabotamp^®^ (0, 33, 55, and 100% coverage of cell layer, respectively) and the fluorescence dye CellTox Green was added to the cells as described in manufacturer’s instructions. The fluorescence signal was measured after 30 min. Total cell lysis was set to 100% cell death. Cell free medium served as background control. The figure shows the means and standard deviations of three independent assays. Statistical analysis was performed with one-way ANOVA followed by Dunnett’s post hoc test. Significance was accepted if *p*-values were <0.05 (* *p* < 0.05, ** *p* < 0.025, *** *p* < 0.001).

Cell death was analyzed via CellTox Green assay following manufacturer’s instructions. The results showed that increasing amounts of Tabotamp^®^ lead to enhanced cell death already after 30 min (Figure 5). At 33% coverage of cell layer, a cell death of 15 ± 1.0% for the Schwann cells, 18 ± 4.7% for immortalized astrocytes, 23 ± 1.0% for neuronal cells, and 39 ± 0.5% for primary astrocytes was determined. With 55% coverage of the cells, Schwann’s cell death increased to 28 ± 3.8%, immortalized astrocytes to 66 ± 10.8%, neuronal cells to 96 ± 5.2%, and primary astrocytes to 60 ± 14.7%, whereas the complete coverage with Tabotamp^®^ led to nearly 100% cell death for all tested cells. Incubation with pH reduced media (pH 6.8, 6.2, 4.8, or 4.2 adjusted by HCl) and measurement of relative amount of death cells after 30 min showed death rates up to 70% (Figure S4).

## 3. Discussion

It is known that ORC reduces pH value dose dependently [13,15,16]. Incubation with pH-reduced medium negatively effects neurite outgrowth [15], dural fibroblast growth [16], and stromal fibroblasts growth and migration rate [13]. Most of these studies have been focused on the effect caused by overall pH reduction, which is thought to be the mechanism of the antimicrobial effect of ORC [17]. In neurosurgery, ORC is used locally to stop diffuse bleeding or to prevent rebleeding after diffuse brain injury via the formation of an artificial coagulum [6,7]. ORC has a typical network structure Figure S3A and in case of surgery, it is placed in a single layer technique on the brain. The question whether there is a local effect of ORC on cerebral tissue has not been analyzed yet.

At first, we determined the pH reduction in PBS, DMEM, and CSF depending on concentration of Tabotamp^®^. In PBS, the strongest and fastest pH reduction was detected. pH values around 2.5 had already been measured with a concentration of 190 mm^2^/mL. Due to their higher protein concentration DMEM and CSF showed a slower pH reduction, but the amount of 500 mm^2^/mL (34 mg/mL) ORC per mL lead to strong acidic pH values of 2.9 for DMEM and 2.8 for CSF. Even a small area of 250 mm^2^/mL caused a pH reduction of CSF to pH value 3.1 (Figure 1). By using EPR, we could confirm the results obtained by the electrode. As shown in Figure 2, the incubation of 250 mm^2^ Tabotamp^®^ caused a reduction of the local pH to 2.2, 2.8, and 3.1 in PBS, DMEM, and CSF, respectively.

A higher reduction of the pH in CSF compared to the cell culture medium was observed, which is caused by higher protein concentration and buffer capacity of the cell culture medium. In comparison to blood plasma, CSF is a body fluid with a low concentration of cells and proteins. Therefore, CSF lacks two buffer systems: high protein content and cellular surface. The only buffering capacity in CSF is the bicarbonate system, which also loses the capacity quickly by releasing CO_2_.

The existence of pH gradients between the ORC (Tabotamp^®^) and the surrounding media is very likely. However, neither the size of the gradient (pH difference) nor the local dimensions (in mm) are known. To solve this analytical problem, we used spectral spatial fluorescence microscopy, which reveals a detailed analysis of pH value changes with an exact spatial resolution (Figure 3A). The spectral analysis of the local volume elements indicated very low pH values at the Tabotamp^®^. Within distance of 1 mm from the Tabotamp^®^ sheet, the pH values increased gradually. At larger distances, no pH gradients were observed. The sensitive pH range for SNARF is from 5 to 7 due to the pK_a_ = 6.4. Outside this range, only a rough estimation of the pH can be made due to the sigmoidal shape of the calibration curve (Figure S1). Below 4.4 and above 7.7 the spectra do not change anymore and the calibration curve is flat. To investigate the local pH at the surface of Tabotamp^®^, additional EPR measurements were performed with the pH-sensitive radical HM with an even lower pK_a_ (Figure 3B). HM offers the possibility to investigate the pH between 2 and 7. Despite the higher sensitivity of the EPR probe to lower pH values, the measured pH values in PBS were again at the lower limit of the sensitivity range, indicating pH values around 2 or even lower.

Further, we incubated the confluent cell layers directly with Tabotamp^®^. We observed a color change to yellow in cell culture medium DMEM due to phenol red, which is part of DMEM as pH indicator (Figure S3B). It could be shown previously that the phenol red indicator has a color change to red at pH values below 1 [18]. In the pH range between 1 and 7, a yellow color is expected. As demonstrated in Figure S3C, the fiber ends of Tabotamp^®^ have been shown a red color, whereas the surrounding area changed the color immediately to yellow after contact to Tabotamp^®^. This observation indicates a local pH reduction effect. The red color of the phenol red medium indicates a pH around 1 at the Tabotamp^®^ surface. This very high acidity was not expected, but it is in accordance with the results of the EPR measurements. The use of two independent methods makes an artifact unlikely.

In previous studies [17], 15 mg/mL had been used to prove the antibacterial effect of Tabotamp^®^. In the present study, cells were covered by 380 mm^2^ Tabotamp^®^ and cultured with 10 mL cell culture medium, corresponding a calculated concentration of 2.58 mg/mL. This concentration of ORC per milliliter DMEM reduced the total pH value from 7.6 to 7.0 (Figure 1). The incubation of cells with pH reduced medium (pH 6.8) showed no relevant cytotoxic effect (Figure S4). Analysis of astrocytes, neurons, Schwann cells, and primary astrocytes by crystal violet staining of the cell layers demonstrated cell damage in the Tabotamp^®^ covered area after an incubation time of 24 h, compared to gauze (Figure 4). In contrast, Tabotamp^®^ had no obvious detectable effect on the surrounding cell layer. Therefore, we concluded that Tabotamp^®^ has a local cytotoxic effect on the covered cells. This hypothesis is supported by prominent subperineural nerve fiber damage caused by local application of ORC on rat sciatic nerve [15].

To confirm the hypothesis of a local cytotoxic effect, CellTox Green assay (Promega) was performed. The results showed a coverage dependent cytotoxic effect of Tabotamp^®^. Interestingly, cytotoxicity was already detectable 30 min after incubation (Figure 5) and the relative amount of dead cells correlated with the area covered by Tabotamp^®^. Nagamatsu also described early changes in subperineural pH values. They found a drop of pH in the first 15 min [15]. Furthermore, the results of cytotoxicity analysis demonstrated different ORC-dependent pH sensitivity of cell types. For neuronal cells, a cell death rate of 96% was detected by 55% Tabotamp^®^ coverage in comparison to Schwann cells, which showed only 28% dead cells. Moreover, the primary astrocytes responded more sensitive to the Tabotamp^®^ (39% cell death by 33% ORC coverage) compared to the immortalized cell line (18% cell death by 33% ORC coverage). Wang et al. described that treatment of primary cultured mouse cortical neurons with pH 6.0 solution (treatment for 1 h and 24 h recovery) leads to an atypical necrotic phenotype [19]. Acidosis can occur in different neurological disorders, e.g., ischemic stroke or neural injury. However, there is no study which investigates the effect of exogenous acidotoxicity on neuronal or glial cells in vitro. Therefore, we incubated the cell layers with pH-reduced media (pH 6.8, 6.2, 4.8, 4.2) to check the direct influence of pH and found a rising relative amount of cell death (Figure S4). The used cell systems are limited in vitro systems, which are not suitable to study the influence of immune response, cell interactions and tissue structure. Therefore, the effects of ORC pH reduction on eloquent cerebral areas should be analyzed in murine models in vivo, prospectively.

## 4. Materials and Methods

### 4.1. ORC Samples

Tabotamp^®^ (Johnson & Johnson Medical, Ethicon, Neuchâtel, Switzerland) is a network of oxidized, regenerated fibers (Figure S2). 500 mm^2^ is equivalent to 34 mg. Tabotamp^®^ is the European equivalent to Surgicel^®^ (Johnson & Johnson Medical, New Brunswick, CA, USA). In the experiments described, Tabotamp^®^ was used as a common example for an ORC.

### 4.2. pH Measurements by pH Electrode

The pH electrode-based measurements were conducted with a HI2020 edge pH meter (Hanna instruments, Vöhringen, Germany) in PBS, supplemented DMEM or CSF at room temperature (Figure 1). Tabotamp^®^ was mixed with the different aqueous solutions for 10 min previous to the pH measurement. After longer incubation periods the pH value did not changed. pH values below 3 are outside of the calibration range of the pH meter. The analysis was carried out in three independent experiments.

### 4.3. Electron Paramagnetic Resonance (EPR) Spectroscopy

The pH-sensitive EPR spin probe 2,2,3,4,5,5-hexamethyl-imidazoline-1-oxyl (HM) [20] with a pK_a_ = 4.7 [21] was obtained from the Institute of Chemical Kinetics and Combustion, Novosibirsk, Russia. Samples were measured in 50 µL capillaries at room temperature with X-band EPR spectrometer at 9.30–9.55 GHz (Miniscope MS 200, Magnettech, Berlin, Germany) at room temperature to be comparable with the pH meter results. The OriginPro version 8.5.1 software (OriginLab, Northampton, MA, USA) was used for spectral analysis. Two Gaussian functions were used to fit the first and the second peak of the spectra. The distance between the two peaks corresponds to the hyperfine splitting a_N_. The pH calibration curve was measured with HM 1 mmol/L in DPBS (Sigma-Aldrich, Steinheim, Germany) and 0.02% Sodium azide (Merck Schuchardt, Hohenbrunn, Germany) in diluted hydrochloride acid. 50 mm^2^ Tabotamp^®^ (3.4 mg) was mixed with 100 µL of PBS, DMEM, or CSF including 1 mmol/L HM or 200 µL, respectively. The experiments with PBS, DMEM, and CSF were carried out in triplicate (Figure 2).

### 4.4. Fluorescence Microscopy

The fluorescence dye SNARF-4F 5-(and-6)-carboxylic acid (SNARF) was purchased from ThermoFisher Scientific. The fluorescence microscope consists of a light source (PhotoFluorII NIR, 89 North, Burlington, VT, USA), a microscope (Leica DM4000B, Leica Microsystems, Wetzlar, Germany) with Nuance EX Fluorescence detector and Nuance Software (PerkinElmer, Hopkinton, MA, USA). Two filter sets for green (515–560 nm) and red (620–660 nm) excitation light were used to illuminate the sample. The emitted light was detected above the excitation wavelength. Multispectral imaging cube sets were acquired in 1 nm steps using automatic exposure times [20]. The intensities at I(609 nm) and I(625 nm) at green excitation light and I(679 nm) at red excitation light were read out of the cube and saved as images, which were loaded in the origin software and handled as matrices. The ratio of intensities was calculated as follows.

$$\mathrm{ratio}=\frac{\left[I{\left(609\;\mathrm{nm}\right)}^{\mathrm{gr}}-I{\left(679\;\mathrm{nm}\right)}^{\mathrm{red}}\right]}{I{\left(625\;\mathrm{nm}\right)}^{\mathrm{gr}}}$$

The pH was calculated with a sigmoidal calibration curve (Figure S1). The calculated pH is shown in Figure 3B. SNARF (21 µg) was dissolved in 200 µL CSF, 23 mm^2^ (1.5 mg) Tabotamp^®^ were placed at a cavity slide and wetted with 50 µL of dye solution.

### 4.5. Fluorescence Imaging

The fluorescence images were made with the Maestro in vivo fluorescence imaging system from Cambridge Research & Instrumentation (Caliper Life Sciences, Waltham, MA, USA) and the Maestro software (version 2.10) with a green (503–555 nm) and yellow (575–605 nm) filter. The measurements were made with and without dye to subtract the background and the auto-fluorescence.

SNARF (12 µg) was dissolved in 2.5 mL of PBS, DMEM or CSF and filled in 5 cm petri dishes. A single layer of Tabotamp^®^ (95 mm^2^ = 6.5 mg) was stamped out and placed in the center of the petri dish. Pictures were made with a Canon 600D camera (Canon Europe, Amstelveen, The Netherlands) on white background. With the Maestro the exposure time was set automatically with the green filter set and used analogue for the yellow filter set. The emission spectra were summed up in one area near the rim and one area near to Tabotamp^®^ as regions of interests (ROI). The measured signals were corrected for background and auto-fluorescence with the manual compute spectra function. Afterwards the spectra of the acidic and neutral areas were unmixed and displayed in the compare image mode. The scale is in parts of the maximum intensity. The neutral and acid areas are sown in in Figure 3A.

### 4.6. Cell Lines

As previously published [22], the murine cell lines C8D1A (CRL-254, astrocytes), RN33B (CRL-2825, neuronal cells), and SW10 (CRL-2766, Schwann cells) were purchased from the American Type Culture Collection (ATCC, Manassas, VA, USA). In brief, astrocytes and Schwann cells were cultured in Dulbecco’s Modified Eagle’s Medium (DMEM) and neuronal cells were cultured in DMEM/F12 (1:1, ThermoFisher Scientific, Darmstadt, Germany). The medium was supplemented with 10% fetal calf serum (FCS; Gibco, ThermoFisher Scientific), 2 mM glutamine (Biochrom AG, Merck, Darmstadt, Germany), 100 U/mL penicillin, and 100 μg/mL streptomycin (ThermoFisher Scientific) during 5% CO_2_ at 37 °C and humidified atmosphere. The SW10 cells were passaged every 2–3 days or astrocytes and neuronal cells every 4–5 days. The passage number of the experimental used cells was below 20. All experiments were carried out in three independent experiments.

### 4.7. Primary Cultures

The preparation of astrocytic primary cultures was carried out as described before [23,24]. Briefly, the Wistar rat neonates (0–2 days) were decapitated and the brains were stored in Hank’s balanced salt solution (HBSS, Gibco, ThermoFisher Scientific). After meninges removal the tissue was digested with 1% trypsin and 500 µg/mL DNase (ThermoFisher Scientific) for 5 min. The resuspended cells were seeded on poly-L-lysine coated cell culture flask (T75, Sarstedt Nümbrecht, Germany). On the next day, the cells were washed with HBSS and fresh medium was added. After 7 days the microglia were removed from astrocyte culture, than the cells were passaged every 4–5 days and used for the experiments to passage number 8. The preparation was in accordance with the Policy on Ethics and the Policy on the Use of Animals in Research as indicated in the directive 2010/63/EU of the European Parliament and of the Council of the European Union on the protection of animals used for scientific purposes and were approved by the local authorities for care and use of laboratory animals (permission number: K11M1).

### 4.8. Crystal Violet Staining

SW10, RN33B, C8D1A cells, and primary astrocytes were grown in cell culture dishes (60 cm^2^, TPP, Trasadingen, Switzerland) to complete confluence and afterwards either 380 mm^2^ gauze or Tabotamp^®^ were placed on the cells. After 24 h, the cell culture dishes were washed two times with ice-cold PBS (ThermoFisher Scientific), fixed for 10 min with 10 mL −20 °C-cold methanol (Sigma-Aldrich, Merck, Darmstadt, Germany), and stained for 10 min with 0.5% (w/v) Crystal violet (Merck) solution containing 25% methanol. After drying, the dishes were scanned (Kyocera ECOSYS M6630cidn, Kyocera Europe, Esslingen, Germany) with 300 dpi. Figure 4 shows one dish out of at least three experiments of each cell line or of primary astrocytes.

### 4.9. Cytotoxicity Measurement

5 × 10^3^ SW10 cells, respectively, 1 × 10^4^ astrocytes or neuronal cells, were seeded in triplicates in black flat 96 well cell culture plates (Greiner Bio-One, Frickenhausen, Germany) and incubated for 24 h. Then, 0, 11, 19, and 34 mm^2^ areas of Tabotamp^®^ (Figure 5) (0, 33, 55, and 100% coverage of cell layer) and the fluorescence dye CellTox-Green (1:1000, Promega, Mannheim, Germany) diluted in 100 µL culture medium were added to the cells. For measurement with pH-reduced media, fluorescence dye was directly diluted in 100 µL media at pH 6.8, 6.2, 4.8, or 4.2 adjusted by HCl (Figure S3). The fluorescence signal was measured after 30 min, 1, 6, and 24 h with Tecan Reader F200PRO (Tecan, Männedorf, Switzerland) at 485_Ex_/525_Em_. Adding lysis buffer (Promega) to untreated cells, resulting in totally lysis, served as positive control (100% cell death). Medium without cells served as background control. The data show the means and standard derivations of three independent biological assays (Figure 5). Statistical analysis was performed with one-way ANOVA followed by Dunnett’s post hoc test (SPSS version 25.0, IBM, Ehningen, Germany). Significance was accepted if *p*-values were ≤0.05.

## 5. Conclusions

The aim of our study was to analyze the effects of Tapotamp^®^ mediated pH alterations to investigate whether Tabotamp^®^ is a suitable hemostat in cerebral eloquent areas. We analyzed pH alteration via three independent methods and found a local pH value reduction, which was much stronger (pH around 1) than expected. In addition, we detected a strong gradient between the ORC and the surrounding media. In summary, cell death was observed in all Tabotamp^®^ covered cell monolayers. Further investigations are needed to prove this effect in vivo. For the clinical practice, we conclude ORC may cause negative local effects on the surrounding tissue. If gross total resection (GTR) is performed close to or within an eloquent area, ORC may not the hemostat of choice. On the other hand, the described local effect might be helpful in oncologic neurosurgery.

## Supplementary Materials

The following are available online at https://www.mdpi.com/1996-1944/13/11/2453/s1, Figure S1: Calibration curve of HM with 1 mmol/L in DPBS and diluted HCl. The pH was adjusted with HCl, Figure S2: Calibration curve of SNARF (20 µg/mL). The pH value was adjusted with NaOH or HCl, Figure S3: Representative cell culture dish with 380 mm^2^ Tabotamp^®^. (A) Knitting structure of Tabotamp^®^ (B) After the addition of cell culture medium DMEM the color of phenol red became yellow in surrounding area of Tabotamp^®^ (C) The outermost area of fibers changed the phenol red color to red, which indicates a pH value below 1, Figure S4: Influence of pH reduced cell culture medium on cell death of Schwann cells and astrocytes.
