# Supplementary material for: Tabotamp®, Respectively, Surgicel®, Increases the Cell Death of Neuronal and Glial Cells In Vitro
Source: Materials (Basel). 2020 May 28;13(11):2453. doi: 10.3390/ma13112453 (PMC7321115; doi:10.3390/ma13112453)
Supplement: Supplementary file 1 [file materials-13-02453-s001.pdf]

materials

Supplementary Materials

Tabotamp®, Respectively, Surgicel®, Increases the Cell Death of Neuronal and Glial Cells In Vitro

Sandra Leisz [1], Marie-Luise Trutschel [2], Karsten Mäder [2], Christian Scheller [1], Christian Strauss [1] and Sebastian Simmermacher [1,*]

[1] Department of Neurosurgery, Medical Faculty, Martin Luther University Halle-Wittenberg, 06120 Halle (Saale), Germany; sandra.leisz@uk-halle.de (S.L.); christian.scheller@uk-halle.de (C.S.); christian.strauss@uk-halle.de (C.S.)
[2] Institute of Pharmacy, Faculty of Biosciences, Martin Luther University Halle-Wittenberg, 06120 Halle (Saale), Germany; marie-luise.trutschel@pharmazie.uni-halle.de (M.-L.T.); karsten.maeder@pharmazie.uni-halle.de (K.M.)
* Correspondence: sebastian.simmermacher@uk-halle.de; Tel.: +49 (0) 345 557 5947; Fax: +49 (0) 345 557 1412

Received: 30 April 2020; Accepted: 26 May 2020; Published: date

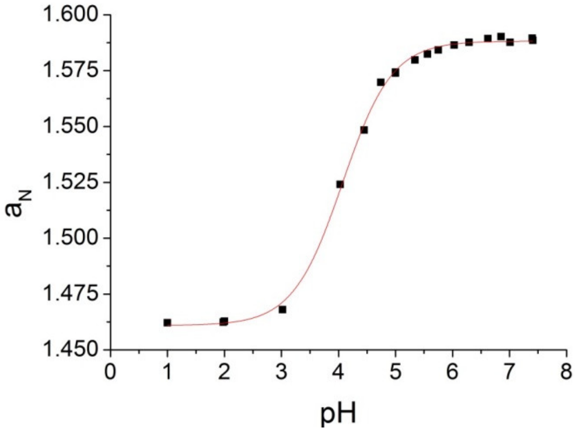

Figure S1. Calibration curve of HM with 1 mmol/l in DPBS and diluted HCl. The pH was adjusted with HCl.

Fluorescence microscopy

The pH values were calculated from the ratio of the intensities. Due to scattering of intensities some ratios for the pH calculation were outside of the calibration curve giving no pH value. To fill the missing picture point with reasonable information, these data points were interpolated with a spline. To reduce the scattering of the pH-value points, the average filter with map padding was used. The amount of pixels was reduced by a factor of four. The calibration curve was made with a dye concentration of 20 µg/mL in phosphate solution. The phosphate solution consists of 16 mg/mL KH_2PO_4 and the pH was changed by the addition of NaOH and HCL solutions (all Grüssing, Filsum, Germany). PBS was made of 0.2 g/L KCl, 8.0 g/l NaCl, 0.19 g/L KH_2PO_4 and 0.588 g/L Na_2HPO_4 (all Grüssing, Filsum, Germany). The fitting function is a sigmoid Boltzmann plot.

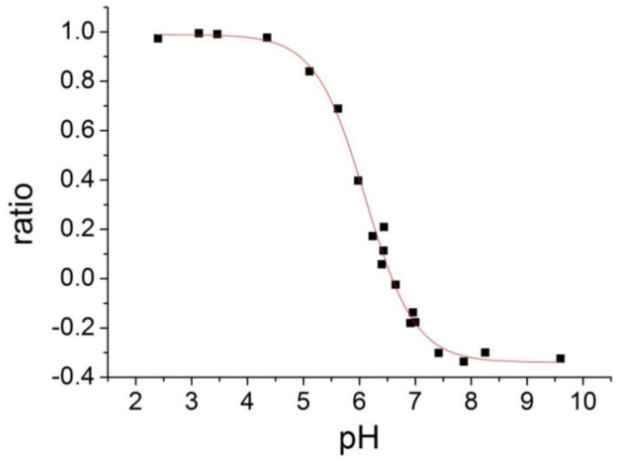

Figure S2. Calibration curve of SNARF-4F (20 µg/mL). The pH value was adjusted with NaOH or HCl.

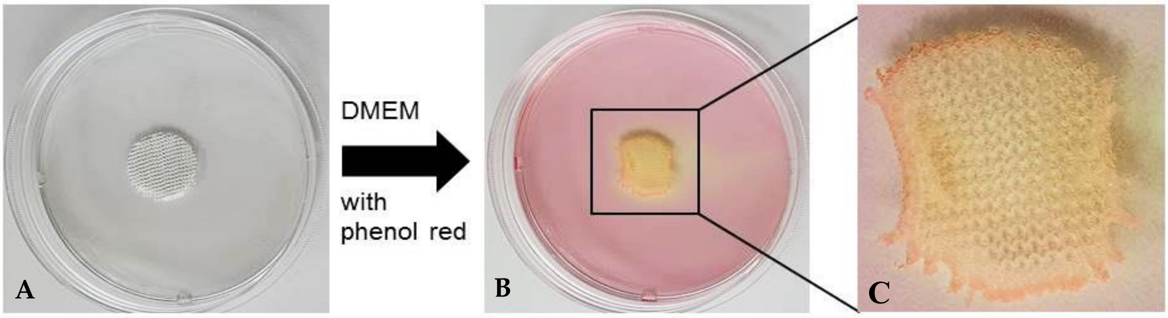

Figure S3. Representative cell culture dish with 380 mm² Tabotamp®. (**A**) Knitting structure of Tabotamp®. (**B**) After the addition of cell culture medium DMEM the color of phenol red became yellow in surrounding area of Tabotamp®. (**C**) The outermost area of fibers changed the phenol red color to red, which indicates a pH value below 1.

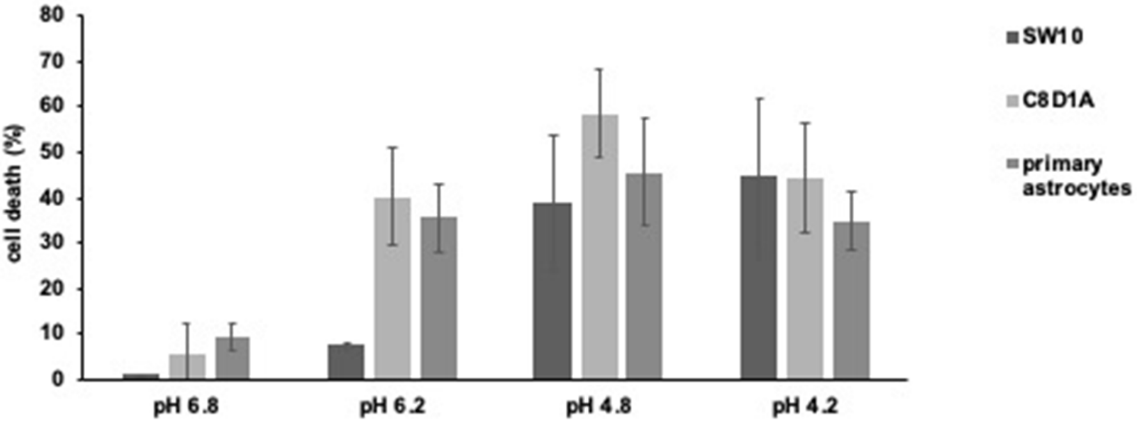

Figure S4. Influence of pH reduced cell culture medium on cell death of Schwann cells and astrocytes.

5 × 10^3 SW10 cells or 1 × 10^4 astrocytes (C8D1A; primary astrocytes) were incubated with pH-reduced media supplemented with CellTox-Green fluorescence dye. The fluorescence signal was measured after 30 min. Total cell lysis was set to 100% cell death. Medium without cells served as background control.
